# IPCT: Integrated Pharmacogenomic Platform of Human Cancer Cell Lines and Tissues

**DOI:** 10.3390/genes10020171

**Published:** 2019-02-22

**Authors:** Muhammad Shoaib, Adnan Ahmad Ansari, Farhan Haq, Sung Min Ahn

**Affiliations:** 1 Department of Biomedical Engineering, College of Medicine, University of Ulsan, Asan Medical Center, Seoul 100-011, Korea; muhemmed.shoaib@gmail.com (M.S.); adnanansa@gmail.com (A.A.A.); 2 Gachon Institute of Genome Medicine and Sciences, Incheon 400-011, Korea; 3 Department of Biosciences, COMSATS University Islamabad, Islamabad 45710, Pakistan; 4 Department of Genome Medicine and Science, College of Medicine, Gachon University, Seongnam 461-140, Korea

**Keywords:** genomics, pharmacogenomics, cell lines, database, drug sensitivity

## Abstract

(1) *Motivation*: The exponential increase in multilayered data, including omics, pathways, chemicals, and experimental models, requires innovative strategies to identify new linkages between drug response information and omics features. Despite the availability of databases such as the Cancer Cell Line Encyclopedia (CCLE), the Cancer Therapeutics Response Portal (CTRP), and The Cancer Genome Atlas (TCGA), it is still challenging for biologists to explore the relationship between drug response and underlying genomic features due to the heterogeneity of the data. In light of this, the Integrated Pharmacogenomic Database of Cancer Cell Lines and Tissues (IPCT) has been developed as a user-friendly way to identify new linkages between drug responses and genomic features, as these findings can lead not only to new biological discoveries but also to new clinical trials. (2) *Results*: The IPCT allows biologists to compare the genomic features of sensitive cell lines or small molecules with the genomic features of tumor tissues by integrating the CTRP and CCLE databases with the REACTOME, cBioPortal, and Expression Atlas databases. The input consists of a list of small molecules, cell lines, or genes, and the output is a graph containing data entities connected with the queried input. Users can apply filters to the databases, pathways, and genes as well as select computed sensitivity values and mutation frequency scores to generate a relevant graph. Different objects are differentiated based on the background color of the nodes. Moreover, when multiple small molecules, cell lines, or genes are input, users can see their shared connections to explore the data entities common between them. Finally, users can view the resulting graphs in the online interface or download them in multiple image or graph formats. (3) *Availability and Implementation*: The IPCT is available as a web application with an integrated MySQL database. The web application was developed using Java and deployed on the Tomcat server. The user interface was developed using HTML5, JQuery v.3.1.0, and the Cytoscape Graph API v.1.0.4. The IPCT web and the source code are available in Sample Availability section.

## 1. Introduction

Advancements in pharmacogenomics through comprehensive next-generation sequencing studies have paved the way for developing effective therapeutics against cancer. The omics data of cancer cell lines and cancer tissues are now readily used for categorizing genomic diversity and identifying anti-cancer drug responses [1]. However, in the era of big data, biologists face new challenges in dealing with the large amount of segregated data available in different cancer genomic repositories [2,3]. 

In the past decade, data scientists have developed numerous biological databases to help biologists analyze the underlying genetic mechanisms of cancer. NCI-60 [2], the first cancer cell line database, remained a unique resource of in vitro drug discovery for many years [4]. Recently, large pharmacogenomic databases such as the Cancer Cell Line Encyclopedia (CCLE) [5], Genomics of Drug Sensitivity in Cancer (GDSC), and the Cancer Therapeutics Response Portal (CTRP) have also emerged. The CCLE provides genomic and transcriptomic information on 947 human cancer cell lines and the drug response data for 24 compounds [5]. The CTRP provides drug response information for more than 860 cancer cell lines against 481 compounds [6]. Furthermore, in addition to cell line data, the omics data of thousands of cancer patients were also generated by The Cancer Genome Atlas (TCGA) and the European Molecular Biology Laboratory (EMBL) [7].

Unfortunately, the volume and heterogeneity of the data has prevented biologists from making effective use of these databases [8]. Therefore, an efficient and biologist-friendly integration of these omics and pharmacogenomics databases is needed. This integration would help biologists generate accurate and practical hypotheses for identifying anti-cancer drug responses. The prime objective of this study was to provide a uniquely user-friendly platform for cancer biologists that they can use to investigate interlinked pharmacogenomics and cancer genomics data.

In this study, we have developed the Integrated Pharmacogenomics Platform of Cancer Cell Lines and Tissues (IPCT), which integrates major drug response information from the CTRP with omics data from the CCLE, cBioPortal [8], REACTOME [9], and Expression Atlas [10] databases. The IPCT is a biologist-friendly platform with numerous novel features, highlighting:

(1) the genomic features sensitive to specific drugs;
(2) the percentage of affected cancer patients sensitive to a drug;
(3) the pathways associated with the drug response;
(4) cancer cell lines that are true representatives of cancer tissues;
(5) user-friendly single-click access to multiple datasets, which facilitates the generation of new and practical hypotheses.

## 2. Materials and Methods

The CTRP contains quantitatively measured sensitivity for 481 small molecules in 860 deeply characterized cancer cell lines. The IPCT (1) integrates the CTRP database with external biological databases and (2) allows biologists to query CTRP data in an integrated graphical fashion. Biologists can start querying by entering a list of cell lines or small molecules and use the context of the results of their search to generate new hypotheses.

The IPCT was developed in three different phases:

(1) construction of the database
(2) development of the database update pipeline
(3) design of the web application

The database is an essential component of the IPCT, storing all the data points and the connections between those data points from the CTRP, CCLE, cBioPortal, REACTOME, and Expression Atlas databases. The update pipeline is a script written in Python that is used to update the database in real time. The web application is a GUI-based application that will be used by the end users to explore the data points and their connections. 

**Figure 1:**
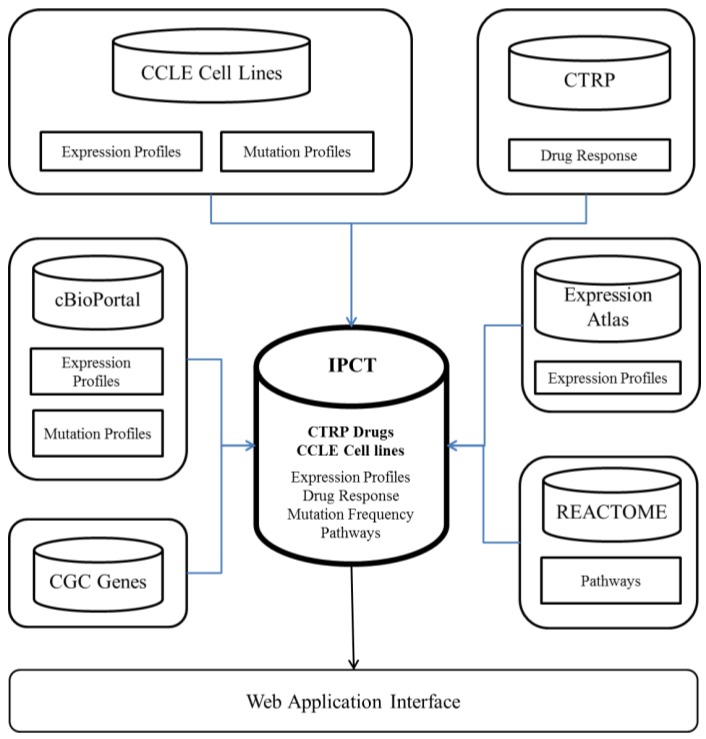
Entities in the IPCT database.

**Figure 2:**
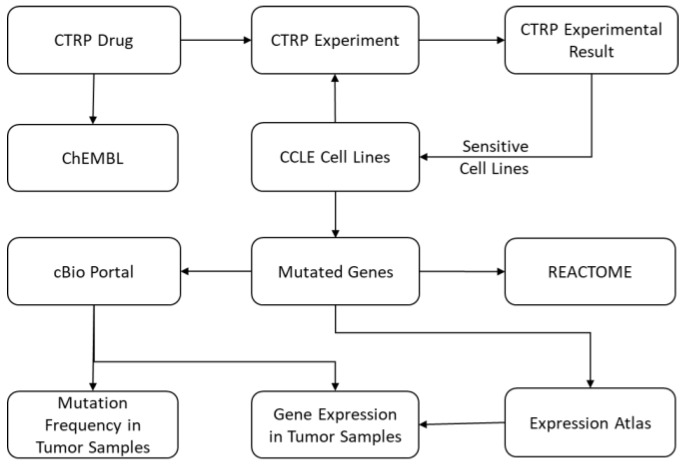
Connectivity map of CTRP drugs and CCLE cell lines with ChEMBL, REACTOME, the Expression Atlas, and cBioPortal.

The IPCT is a biological database that integrates data about cancer cell lines, small molecules, human pathways, experimental results, and cancer somatic mutations. Figure 1 and Figure 2 show the architecture of the IPCT database and demonstrate how multiple databases have been integrated in the IPCT database. We collected the cell line data from the CCLE dataset, the small molecule features from the CTRP dataset, the pathway data from REACTOME, the expression data from the Expression Atlas, the list of cancer genes from cancer genes census [11] and OncoKB [12], and the genomic features of cancer studies from cBioPortal. Our objective was to create an integrated database by connecting the data points in the above databases. 

The CTRP database contains experimental details and results reported as the area under the curve (AUC). Therefore, as a first step, we computed a sensitivity score for each small molecule against each cell line using these AUC values. The sensitivity scores were computed using the R package “extremevalues” in a similar manner to Speyer et al. [13]. After this step, we obtained a numeric score for each small molecule–cell line pair. A small molecule is sensitive to a cell line if its sensitivity score is below −1 and resistant if its sensitivity score is above 1 [13]. This sensitivity score was then used to construct a small molecule–cell line network, and the small molecules were connected to the ChEMBL database. A total of 158 small molecules were common between the CTRP and ChEMBL.

In the second step, we added genes to our network. To do this, a list of genes and their relationships to cell lines was required. We extracted genetic metadata from the NCBI website and connected the genes and cell lines based on genomic changes, which were present in the CCLE dataset in the form of mutations, copy number alterations, and gene expression. We extracted these for each cell line from the CCLE dataset and used this information to construct a small molecule–cell line–gene graph. A gene was included in the small molecule–cell line–gene network if it had mutations, copy number amplification, copy number deletion, high expression, or low expression in at least 10% of the cell lines sensitive to an input small molecule. The IPCT, by default, connects only genes with genomic aberration in 20% of the cell lines sensitive to an input small molecule. However, users can relax or tighten these criteria as needed. Having already constructed a small molecule–cell line network, in this step we only had to connect the cell lines with the genes. To do this, we connected cell lines with the genes that had mutations or copy number alterations in the given cell lines. This process was repeated for all cell lines in the CCLE, which resulted in a cell line–gene network with genes and cell lines as nodes and mutations or copy number alterations as edges. The cell line–gene graph was then merged with the small molecule–cell line graph, which resulted in a small molecule–cell line–gene network. After this step, we could identify genes with mutations or copy number alterations in the cell lines that are sensitive to a given set of small molecules. 

Once we had identified the mutated genes, we added the pathways of the mutated genes to our small molecule–cell line–gene network, collecting pathway data from the REACTOME database. These pathways were connected using Entrez GeneIDs present in both databases.

The next step was to identify if the mutated genes had been reported as up-regulated or down-regulated in previous experiments. The Expression Atlas contains differential expression data from approximately 2500 experiments performed in different experimental conditions. However, the Expression Atlas uses Ensembl IDs instead of gene names or Entrez GeneIDs in its analyzed files. In the first step, we filtered only those experiments that were related to cancer, loaded them into the database, and removed insignificant records with *p*-value > 0.05 and log fold change >−1 and <1. Records with log fold change ≥1 or ≤−1 and *p*-value < 0.05 were used for further processing. Next, we connected all Ensembl IDs with their Entrez GeneIDs using the R package “org.Hs.eg.db”. We used this database to construct a gene–experiment network with genes and experiments as nodes and up-regulation or down-regulation as edges. This graph was then merged with the small molecule–cell line–gene network constructed in the previous step.

After construction of this pathway graph, our next task was to identify if the mutated genes had any potential relationship with any cancer types in published cancer studies. To do this, we extracted data from cBioPortal. For each gene, we computed what percentage of samples were mutated, altered, up-regulated, and down-regulated in each study, identifying mutation and copy number alteration frequencies for 30,000 genes in 151 cancer studies and 33 cancer types. The data from cBioPortal were not used in network construction but are available as a separate entity for further investigation. 

The IPCT can be accessed via the web application, which allows users to explore the connections between the data points of five biological databases in an integrated graphical fashion. When a user enters a small molecule, cell line, or gene, a graph is displayed with the data points as nodes and the relationships between the data points as edges. Using this graph, the user can intuitively investigate the connectivity of the given small molecules, cell lines, and genes. When a user enters multiple cell lines, small molecules, or genes, the IPCT first independently constructs a graph for each element in the list. Next, it takes two random graphs from among those and merges them using the common data points. This step is repeated until all the graphs are merged into one graph, which is ultimately displayed to the user. 

## 3. Results

The IPCT comprises two major components: (1) the IPCT database and (2) the IPCT web portal. The IPCT web portal provides an easy way to investigate the connections between the data points available in the CTRP, CCLE, Expression Atlas, REACTOME, and cBioPortal databases in an integrated fashion. The IPCT database currently contains 860 cell lines, 481 small molecules, ~2500 differential expression studies, 2000 human pathways, and 151 cancer studies. Moreover, the IPCT contains 8,214,573 unique connections between the different data points (Table S1). The overall database size is 20 GB. The distinctive functionality and features of the IPCT are as follows:

1. Users can input up to ten cell lines, small molecules, or genes to find potential connectivity with other data points.
2. Users can filter small molecules and cell lines sensitive to each other according to a minimum sensitivity score.
3. Users can apply a filter on genes if they want to view only cancer genes, exclude commonly mutated genes, or view all genes.
4. Users can apply filters if they want to see only mutated, copy number altered, or high- or low-expressed genes.
5. Users can check the mutation frequencies and differential expression frequencies in different cancer studies.
6. Users can highlight genes of their interest by applying a gene filter to the network.
7. Users can select if they want to show all connections or only shared connections when multiple cell lines, small molecules, or genes are entered.
8. Users can view the output in the web browser as a graph or table. Alternatively, users can download the graph and view it with Cytoscape version 1.0.4 or graph viewing tools that show JSON and CSV files.
9. Users can save the graphs in JSON, PNG, or PDF formats and table in CSV format.

### 3.1. Data Exploration

**Figure 3:**
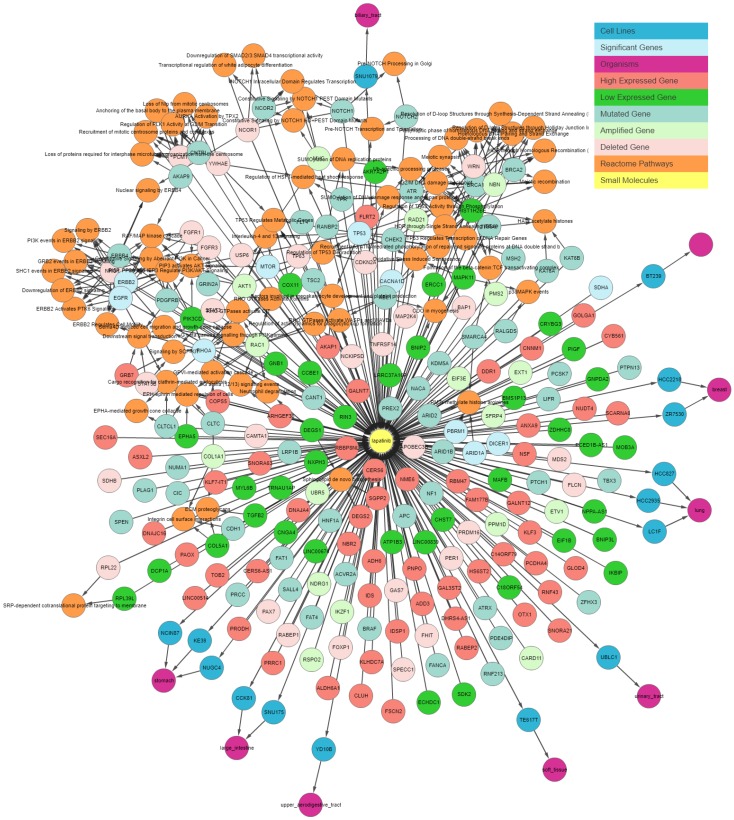
IPCT output for small molecule user query lapatinib. The graph shows all data points connected with lapatinib. Yellow nodes represent small molecules; blue nodes show cell lines sensitive to lapatinib; sky-blue nodes represent significant genes (those with multiple genomic aberrations); green and red nodes represent genes that are up-regulated and down-regulated in the sensitive cell lines, respectively; light green and light red represent the amplified and deleted genes in the sensitive cell lines, respectively; white nodes represent mutated genes; and orange nodes represent the REACTOME pathways of mutated genes.

Users can start exploring the IPCT by entering small molecules, cell lines, or genes. If users enter a list of cell lines, the IPCT outputs graphs with small molecules that are sensitive to the queried cell lines and genes that are mutated or altered in the given cell lines. If users enter a small molecule, the IPCT outputs a graph containing the cell lines sensitive to the given small molecule and genes mutated in the sensitive cell lines. If users enter genes, the IPCT outputs a graph of cell lines with mutations or copy number alterations in the given genes and the small molecules sensitive to those cell lines. Users can then expand their search by expanding the graph to include data points from the Expression Atlas or REACTOME. User can apply filters as explained in Table 1 and reduce number of entities in graph. Figure 3 illustrates the output generated by the IPCT for lapatinib with the shared pathway filter. By default, the IPCT shows the pathways associated with more than 20% of genes connected with the input drug, but users can modify this option to show all pathways if they want to see the pathways of connected genes. Supplementary Figure S1 shows the result of same query with all pathways. The IPCT also allows users to apply different filters to define the context of their search.

### 3.2. Comparison Between Cell Lines and Real Tissues

**Figure 4:**
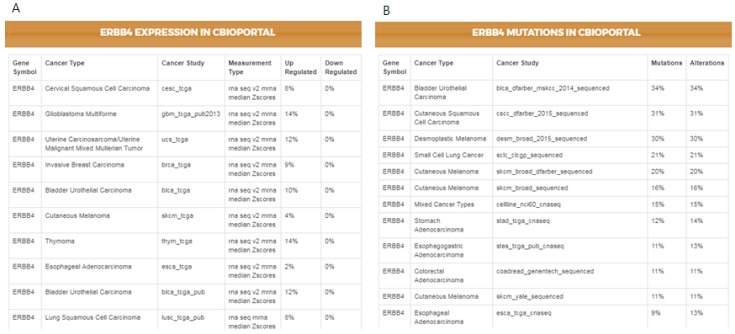
ERBB4’s genetic profile in real tumors extracted from cBioPortal. (**A**) ERBB4’s differential expression in different cancer studies. (**B**) ERBB4’s mutation and copy number alteration frequency in different cancer studies.

As not all cancer cell lines have equal values to the tumor models, comparison between the genetic profiles of cell lines and real tumors is of importance. For example, when a mutation is found in a cell line, the first question might be if the specific mutation has also been reported in any cancer studies, followed by whether the given gene has any reported differential expression. The IPCT, by integrating data from cBioPortal and the Expression Atlas, provides answers to both questions. When a user clicks on a mutated gene’s node, he can explore the cancer studies in which the given gene is up-regulated or down-regulated and observe its mutation or copy number alteration percentages in all cBioPortal cancer studies. For example, in the previously illustrated query (Figure 3), by investigating lapatinib, sorafenib, gefitinib, and sunitinib together, we identified that *ERBB4* is mutated in 38% of cell lines sensitive to sorafenib, 33% of cell lines sensitive to gefitinib, 31% of cell lines sensitive to sunitinib, and 27% of cell lines sensitive to lapatinib. Users could then further investigate its frequency in real tumors. Figure 4 illustrates the results for the mutations and the differential expression frequency of *ERBB4* in different cancer studies. 

### 3.3. Filtering Genes

Cell lines harbor mutations in many genes; however, not all mutated genes are of interest to biologists, who mostly value mutations in oncogenes or tumor suppressor genes because of their well-defined role in cancer. In light of this, the IPCT gene filter has the following three options:

1. Cancer genes: Construct graphs in the context of only oncogenes or tumor suppressor genes.
2. Exclude commonly mutated genes: Construct graphs in the context of all genes, but exclude genes that have mutations in more than 90% of cell lines.
3. All genes: Disable the filter and construct graphs in the context of all genes.

**Figure 5:**
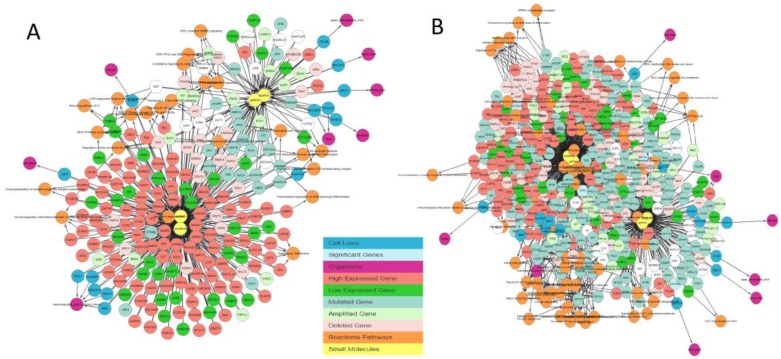
IPCT output for small molecule user query lapatinib, sorafenib, gefitinib, and sunitinib after disabling the REACTOME and Expression Atlas databases and enabling cell lines and mutated genes only. (**A**) Gene filter = only cancer genes. (**B**) Gene filter = exclude common mutations.

This filter enables users to focus on mutations in cancer genes and further explore only cancer genes as well as to focus on rarely mutated genes by allowing them to exclude genes that are mutated in more than 90% of cell lines. Figure 4 illustrates the effect of applying a gene filter. Figure 5A shows only cancer genes that are mutated in sensitive cell lines, and Figure 5B shows the network with all genes excluding frequently mutated genes, i.e., genes that are mutated in more than 90% of overall cell lines. 

### 3.4. Finding Shared Connections

Another important feature of the IPCT for biologists is identifying shared connections between data entities, such as hidden direct or indirect relationships between two cell lines and small molecule sensitivity or between small molecule sensitivity and gene mutations. 

The shared connection filter in the IPCT allows users to investigate unknown or hidden relationships between the data entities of the five connected databases. For example, when a user inputs more than two small molecules, the IPCT constructs a graph with cell lines sensitive to the input small molecules, their mutated genes, and the data entities connected with the mutated genes. By enabling the shared connection filter, users can restrict the results to cell lines sensitive to both small molecules. Similarly, users can restrict the graph to genes mutated in more than one cell line or to pathways that are common between mutated genes.

### 3.5. Case Study

**Figure 6:**
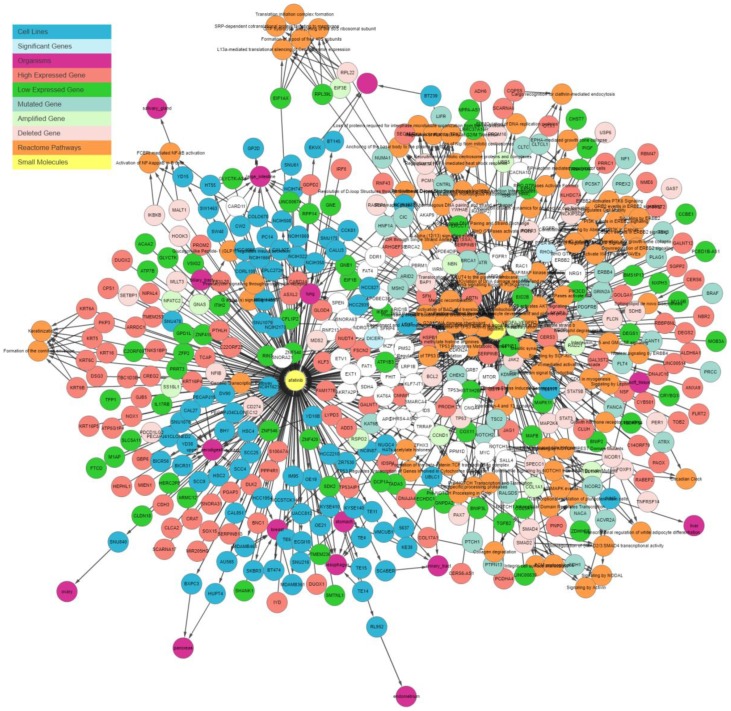
IPCT output for small molecule user query lapatinib and afatinib. The graph shows all data points connected with lapatinib and afatinib. Yellow nodes represent small molecules; blue nodes show cell lines sensitive to lapatinib and afatinib; sky-blue nodes represent significant genes (those with multiple genomic aberrations); green and red nodes represent genes that are up-regulated and down-regulated in the sensitive cell lines, respectively; light green and light red represent the amplified and deleted genes in the sensitive cell lines, respectively; white nodes represent mutated genes; and orange nodes represent the REACTOME pathways of mutated genes.

Lapatinib and afatinib are two tyrosine kinase inhibitors that are effective in breast cancer. These drugs are usually effective in *HER2* (*ERBB2*) mutation-positive patients [14,15]. In this section, we demonstrate how the IPCT can be used to identify the mechanism of action of these two kinase inhibitors. For this purpose, in the first stage, we query lapatinib and afatinib in the IPCT. Figure 6 shows the graph containing the sensitive cell lines, associated genes, and their pathways generated by the IPCT as result of the query, without applying any filter. Genes associated with these drugs are colored and shaped based on their relationship; each color and shape represent a unique relationship between the genes and the cell lines sensitive to the input drug. As such, genes with certain colors and shapes can be classified as more important than other genes.

**Figure 7:**
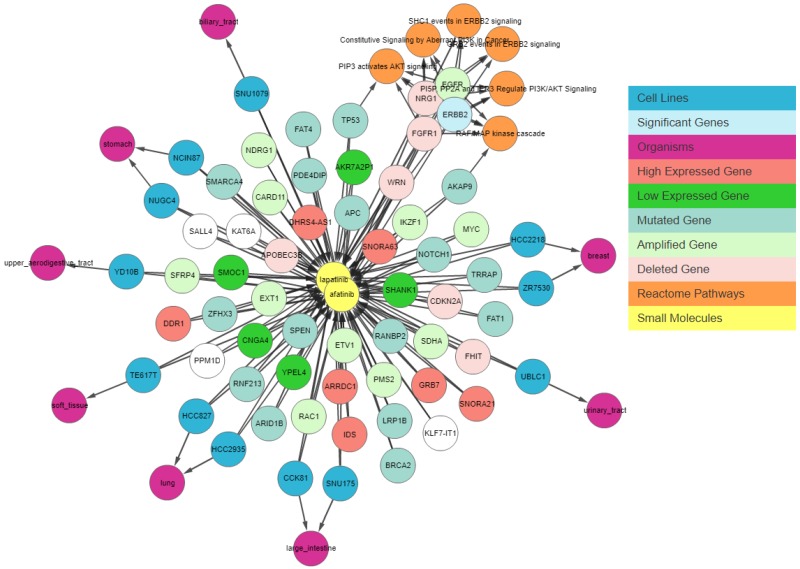
IPCT output for small molecule user query lapatinib and afatinib with the shared connection filter enabled. The graph shows all data points connected with lapatinib and afatinib. Yellow nodes represent small molecules; blue nodes show cell lines sensitive to lapatinib and afatinib; sky-blue nodes represent significant genes (those with multiple genomic aberrations); green and red nodes represent genes that are up-regulated and down-regulated in the sensitive cell lines, respectively; light green and light red represent the amplified and deleted genes in the sensitive cell lines, respectively; white nodes represent mutated genes; and orange nodes represent the REACTOME pathways of mutated genes.

**Figure 8:**
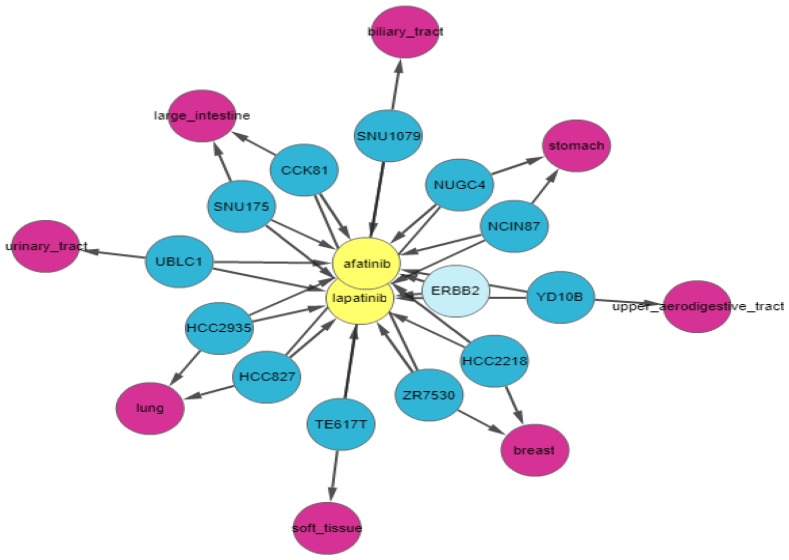
IPCT output for small molecule user query lapatinib and afatinib with the shared connection filter and the relationship filter enabled. The graph shows all data points connected with lapatinib and afatinib. Yellow nodes represent small molecules; blue nodes show cell lines sensitive to lapatinib and afatinib; and sky-blue nodes represent significant genes (those with multiple genomic aberrations).

Next, we apply a filter to shortlist our gene set. We first apply the shared connection filter to see if any genes are associated with both drugs. Genes can have different associations with each drug, and the more important genes will be those that have the same association with both drugs. Figure 7 shows the resulting graph. The sky-blue genes are the most important ones, whereas those with a white background are the least important. The circled genes can be classified as the most relevant gene set due to the pathway clusters. Figure 7 shows that *EGFR* is amplified, *NRG1* and *FGFR1* are deleted, and *AKAP9* and *TP53* have mutations in cell lines sensitive to both drugs. These genes have been found to be relevant to lapatinib and afatinib in the literature [15,16,17]. *ERBB2* is amplified and highly expressed in 95% of afatinib-sensitive cell lines and 100% of lapatinib-sensitive cell lines. Finally, we apply relationship filters to identify the most relevant results. These filters are designed to filter genes that have multiple genomic aberrations with the queried drugs. Finally, Figure 8 demonstrates the relationship of *ERBB2* with lapatinib and afatinib. 

### 3.6. Download Graph

The web interface of the IPCT allows users to navigate the data entities connected with CTRP small molecules or CCLE cell lines. In addition to this, users can also download the output connectivity maps for future reference as image or PDF files. Furthermore, users can also download connectivity maps in the GraphML version 1.0, JSON, PNG, PDF or CSV formats for further analysis or generate high-quality graphs using external graph-making tools such as Cytoscape. In these formats, the nodes represent data entities and the edges represent the connections between them. Each edge is identified with a unique identifier and contains information in the form of a label, database identifier, and URL. The labels store the display information, and the database identifiers store the color coding. Nodes representing small molecules contain additional information about their sensitivity to the connected cell lines. Similarly, nodes representing genes contain additional information about their mutation frequency in the sensitive cell lines. 

## 4. Discussion and Conclusions

Recent advancements in pharmacogenomics through high-throughput sequencing have necessitated that big-data scientists should develop innovative strategies to deal with the rapidly increasing amount of available biological data. The two major limitations to making effective use of all this information are the extensive heterogeneity of the data and a lack of integration. To overcome these limitations, data scientists have developed integrated, biologist-friendly databases. For instance, the European Bioinformatics Institute’s RDF platform is a state-of-the-art example that has enabled the integration of six different biological databases, including UniProt, the Expression Atlas, REACTOME, ChEMBL, BioModels, and BioSamples. However, to the best of our knowledge, no large-scale efforts have been made to integrate the pharmacogenomic features of cancer cell lines with the cancer-related genomic features of real cancer patients.

One of the key questions for any biologist is whether the genomic features of cancer cell lines that are sensitive to drugs are also relevant in real cancer tissues. To address this, biologists previously had to search through multiple heterogeneous databases, which is a challenging job even for researchers with advanced computer skills. Recently, Elena Piñeiro-Yáñez et al. [18] developed PanDrugs to prioritize anticancer drug treatments depending on patients’ genomic profiles. PanDrugs mostly focuses on the clinical aspects of cancer genomics, whereas the IPCT is designed to help researchers in the generation and in silico testing of hypotheses on the pharmacogenomics data of human cell lines and the genomic data of human tumor samples. The IPCT enables data integration and interoperability between the CTRP, CCLE, Expression Atlas, REACTOME, and cBioPortal databases, allowing users to investigate the connectivity maps of cell lines, small molecules, and genes of interest in a user-friendly fashion.

In summary, the IPCT enables biologists to investigate the connectivity of small molecules and genomics features in relationship with cancer cell lines and real cancer tissues. It also highlights the genomic features sensitive to a specific drug and the percentage of cancer patients affected by that drug. Notably, IPCT can also identify cancer cell lines that are truly representative of real cancer tissues. In conclusion, the integration of these five major databases in a biologist-friendly manner will help researchers generate new and tangible hypotheses, leading to further clinical trials in the quest for better cancer treatment.

## Figures and Tables

**Table 1:** Database filters that can be applied to searches in the IPCT.

Database Filter	Applicable Object	Function
Compound sensitivity	Small molecules	Allow users to set thresholds for small molecule sensitivity
Mutation frequency	Genes	Allow users to set mutation frequency
Gene filter	Genes	Allow users to show only cancer genes, exclude commonly mutated genes, or see all genes
Pathway filter	REACTOME	Allows users to select metabolic and signaling pathways
Genomic aberration	Genes	Allow users to filter gene relationships based on mutations, copy number alterations, and gene expression

## Supplementary Materials

The following are available online at https://www.mdpi.com/2073-4425/10/2/171/s1, Figure S1: IPCT output for small molecule user query lapatinib with all pathways levels, Table S1: User Input query for Figure 3, Table S2: User Input query for Figure 4, Table S3: User Input query for Figure 5, Table S4: No of Connections in IPCT, Table S5: Comparison with other Tools.

## References

[1] Gillet J.-P., Varma S., Gottesman M.M. (2013). The clinical relevance of cancer cell lines. JNCI J. Natl. Cancer Inst..

[2] Shoemaker R.H. (2006). The NCI60 human tumour cell line anticancer drug screen. Nat. Rev. Cancer.

[3] Stransky N., Ghandi M., Kryukov G.V., Garraway L.A., Lehár J., Liu M., Sonkin D., Kauffmann A., Venkatesan K., Edelman E.J. (2015). Pharmacogenomic agreement between two cancer cell line data sets. Nature.

[4] Weinstein J.N. (2006). Spotlight on molecular profiling: “Integromic” analysis of the NCI-60 cancer cell lines. Mol. Cancer Ther..

[5] Barretina J., Caponigro G., Stransky N., Venkatesan K., Margolin A.A., Kim S., Wilson C.J., Lehár J., Kryukov G.V., Sonkin D. (2012). The Cancer Cell Line Encyclopedia enables predictive modelling of anticancer drug sensitivity. Nature.

[6] Basu A., Bodycombe N.E., Cheah J.H., Price E.V., Liu K., Schaefer G.I., Ebright R.Y., Stewart M.L., Ito D., Wang S. (2013). An interactive resource to identify cancer genetic and lineage dependencies targeted by small molecules. Cell.

[7] Tomczak K., Czerwińska P., Wiznerowicz M. (2015). The Cancer Genome Atlas (TCGA): An immeasurable source of knowledge. Contemp. Oncol..

[8] Gao J., Aksoy B.A., Dogrusoz U., Dresdner G., Gross B., Sumer S.O., Sun Y., Jacobsen A., Sinha R., Larsson E. (2013). Integrative analysis of complex cancer genomics and clinical profiles using the cBioPortal. Sci. Signal..

[9] Croft D., Mundo A.F., Haw R., Milacic M., Weiser J., Wu G., Caudy M., Garapati P., Gillespie M., Kamdar M.R. (2014). The Reactome pathway knowledgebase. Nucleic Acids Res..

[10] Petryszak R., Burdett T., Fiorelli B., Fonseca N.A., Gonzalez-Porta M., Hastings E., Huber W., Jupp S., Keays M., Kryvych N. (2014). Expression Atlas update—A database of gene and transcript expression from microarray- and sequencing-based functional genomics experiments. Nucleic Acids Res..

[11] Futreal P.A., Coin L., Marshall M., Down T., Hubbard T., Wooster R., Rahman N., Stratton M.R. (2004). A census of human cancer genes. Nat. Rev. Cancer.

[12] Chakravarty D., Gao J., Phillips S., Kundra R., Zhang H., Wang J., Rudolph J.E., Yaeger R., Soumerai T., Nissan M.H. (2017). OncoKB: A precision oncology knowledge base. JCO Precis. Oncol..

[13] Speyer G., Mahendra D., Tran H.J., Kiefer J., Schreiber S.L., Clemons P.A., Dhruv H., Berens M., Kim S. (2016). Differential pathway dependency discovery associated with drug response across cancer cell lines. Pa. Symp. Biocomput..

[14] Rimawi M.F., Aleixo S.B., Rozas A.A., Nunes de Matos Neto J., Caleffi M., Figueira A.C., Souza S.C., Reiriz A.B., Gutierrez C., Arantes H. (2015). A neoadjuvant, randomized, open-label phase II trial of afatinib versus trastuzumab versus lapatinib in patients with locally advanced HER2-positive breast cancer. Clin. Breast Cancer.

[15] Li D., Ambrogio L., Shimamura T., Kubo S., Takahashi M., Chirieac L.R., Padera R.F., Shapiro G.I., Baum A., Himmelsbach F. (2008). BIBW2992, an irreversible EGFR/HER2 inhibitor highly effective in preclinical lung cancer models. Oncogene.

[16] Forster J.A., Paul A.B., Harnden P., Knowles M.A. (2011). Expression of NRG1 and its receptors in human bladder cancer. Br. J. Cancer.

[17] Leech A.O., Vellanki S.H., Rutherford E.J., Keogh A., Jahns H., Hudson L., O’Donovan N., Sabri S., Abdulkarim B., Sheehan K.M. (2018). Cleavage of the extracellular domain of junctional adhesion molecule-A is associated with resistance to anti-HER2 therapies in breast cancer settings. Breast Cancer Res..

[18] Piñeiro-Yáñez E., Reboiro-Jato M., Gómez-López G., Perales-Patón J., Troulé K., Rodríguez J.M., Tejero H., Shimamura T., López-Casas P.P., Carretero J. (2018). PanDrugs: A novel method to prioritize anticancer drug treatments according to individual genomic data. Genome Med..

