# Supplementary material for: IPCT: Integrated Pharmacogenomic Platform of Human Cancer Cell Lines and Tissues
Source: Genes (Basel). 2019 Feb 22;10(2):171. doi: 10.3390/genes10020171 (PMC6409836; doi:10.3390/genes10020171)
Supplement: Supplementary file 1 [file genes-10-00171-s001.pdf]

Supplementary Information

IPCT: Integrated Pharmacogenomic platform of cancer Cell lines and Tissues

Muhammad Shoaib[1.2], Adnan Ahmad Ansari[1,2], Farhan Haq[4,*], and Sung Min Ahn[2,3, *]

[1]Department of Biomedical Engineering, College of Medicine, University of Ulsan, Asan Medical Center, Seoul, Republic of Korea, [2]Gachon Institute of Genome Medicine and Sciences and [3]Department of Genome Medicine and Science, College of Medicine, Gachon University, Seongnam, Republic of Korea, [4]Department of Biosciences, COMSATS University Islamabad Pakistan

*To whom correspondence should be addressed.

Muhammad Shoaib
2016-06-23

IPCT User Queries

In the following supplementary tables, we have presented details about queries that we inputted to the IPCT for generation of figure 1, 3 and 4. Readers can use the information given in the following tables to regenerate the similar graphs as given in the paper.

Table S1: User Input query for Figure 1

<table>
<tr><td colspan="2">Input Type</td><td colspan="3">Input</td></tr>
<tr><td colspan="2">Small Molecule</td><td colspan="3">LAPATINIB, SORAFENIB, GEFITINIB and SUNITINIB</td></tr>
<tr><td colspan="2">Databases Included</td><td colspan="3">Associated Genes, Cell lines, Expression Atlas, REACTOME</td></tr>
<tr><td colspan="2">Databases Excluded</td><td colspan="3">None</td></tr>
<tr><td colspan="2">Genes Filter</td><td colspan="3">Cancer Genes Only</td></tr>
<tr><td colspan="5">Filters</td></tr>
<tr><td>Drug Sensitivity</td><td>Mutation Frequency</td><td>Pathway Filter</td><td>Graph Type</td><td>Output Type</td></tr>
<tr><td>-1.50</td><td>20%</td><td>All Pathways</td><td>All Connections</td><td>View Graph</td></tr>
</table>

Table S2: User Input query for Figure 3

<table>
<tr><td colspan="2">Input Type</td><td colspan="3">Input</td></tr>
<tr><td colspan="2">Small Molecule</td><td colspan="3">LAPATINIB, SORAFENIB, GEFITINIB and SUNITINIB</td></tr>
<tr><td colspan="2">Databases Included</td><td colspan="3">Associated Genes, Cell LInes,</td></tr>
<tr><td colspan="2">Databases Excluded</td><td colspan="3">Expression Atlas, REACTOME</td></tr>
<tr><td colspan="2">Genes Filter</td><td colspan="3">a) Cancer Genes Only
b) All Genes</td></tr>
<tr><td colspan="5">Filters</td></tr>
<tr><td>Drug Sensitivity</td><td>Mutation Frequency</td><td>Pathway Filter</td><td>Graph Type</td><td>Output Type</td></tr>
<tr><td>-1.50</td><td>20%</td><td>All Pathways</td><td>All Connections</td><td>View Graph</td></tr>
</table>

Table S3: User Input query for Figure 3

<table>
<tr><td colspan="2">Input Type</td><td colspan="3">Input</td></tr>
<tr><td colspan="2">Small Molecule</td><td colspan="3">LAPATINIB, SORAFENIB, GEFITINIB and SUNITINIB</td></tr>
<tr><td colspan="2">Databases Included</td><td colspan="3">Associated Genes, Cell LInes, Expression Atlas, REACTOME</td></tr>
<tr><td colspan="2">Databases Excluded</td><td colspan="3">None</td></tr>
<tr><td colspan="2">Genes Filter</td><td colspan="3">Cancer Genes Only</td></tr>
<tr><td colspan="5">Filters</td></tr>
<tr><td>Drug Sensitivity</td><td>Mutation Frequency</td><td>Pathway Filter</td><td>Graph Type</td><td>Output Type</td></tr>
<tr><td>-1.50</td><td>20%</td><td>All Pathways</td><td>Common Connections</td><td>View Graph</td></tr>
</table>

Table S4: No of Connections in IPCT

Connection Type	No of Connections
Drug Gene Connections	30,340
Mutations	1,683,111
Copy Number Alterations	1,528,174
Amplifications	648,471
Deletions	879,703
Gene Expression	1,610,795
High Expression	949,031
Low Expression	661,764
Gene-Pathway	223,184
Total	8,214,573

Table S5: Comparison with other Tools

Database	Genomic Aberrations	Drug Sensitivity	Cell Line and Tissue Comparisons	Networked View
CCLE	Yes	No*	No	No
CTRP	No	Yes	No	Yes
GDSE	No	Yes	No	No
IPCT	Yes	Yes	Yes	Yes

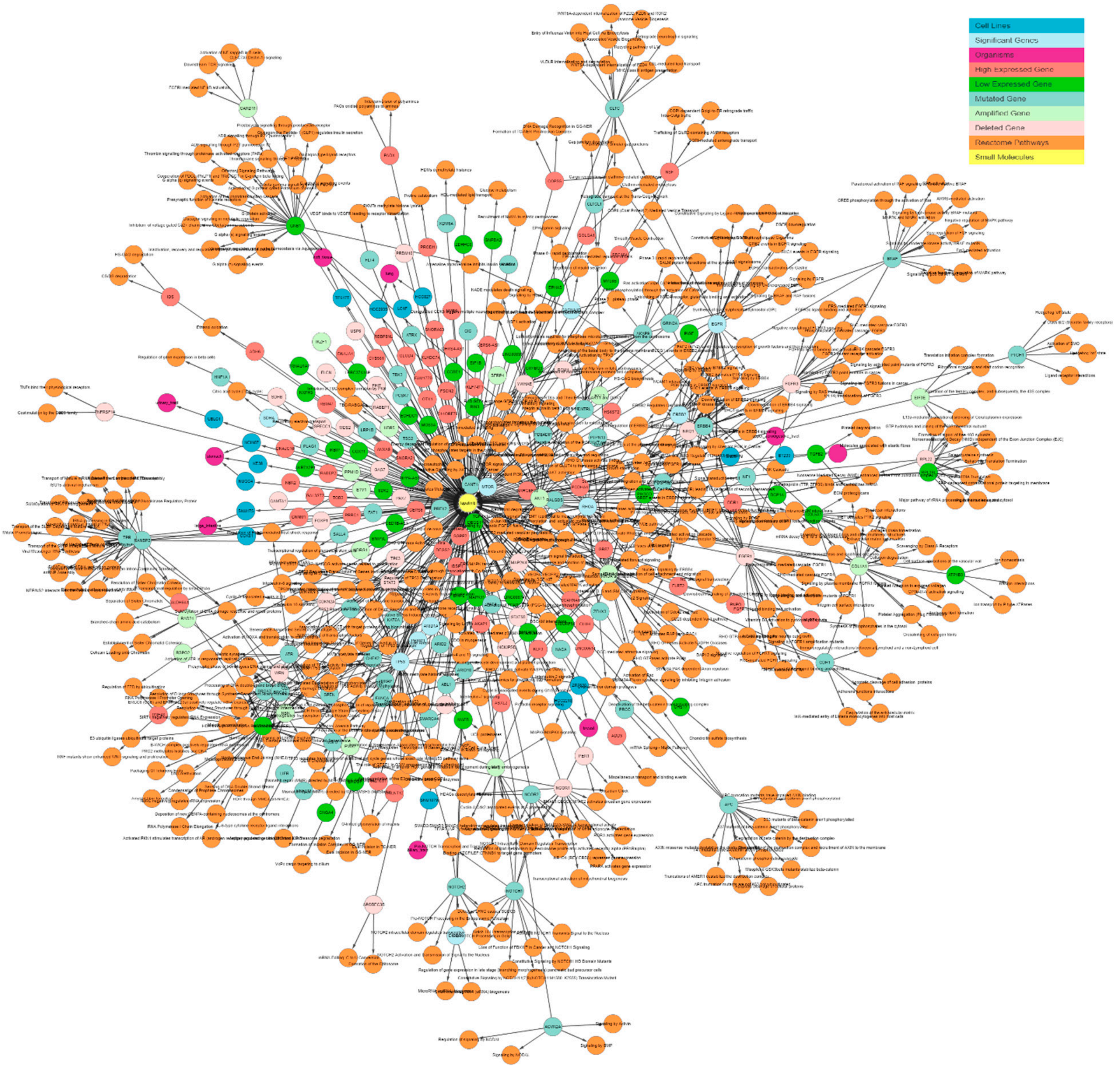

Figure S1: *IPCT output for small molecule user query lapatinib with all pathways levels. The graph shows all data points connected with lapatinib. Yellow nodes represent small molecules; blue nodes show cell lines sensitive to lapatinib; sky-blue nodes represent significant genes (those with multiple genomic aberrations); green and red nodes represent genes that are upregulated and downregulated in the sensitive cell lines, respectively; light green and light red represent the amplified and deleted genes in the sensitive cell lines, respectively; white nodes represent mutated genes; and orange nodes represent the REACTOME pathways of mutated genes.*

Supplementary Case Study:

Dabrafenib and trametinib are BFAF inhibitors that are effective in melanoma skin cancer. These drugs are usually effective in BFAF mutation-positive patients. In Supplementary figure S2, we demonstrate how the IPCT can be used to identify the important gene i.e. BRAF that is associated with dabrafenib and trametinib. We have applied shared connection filter that gave us graph containing all genes that has some genomic association with cell lines sensitive to dabrafenib and trametinib. and highlight gene using gene find function.

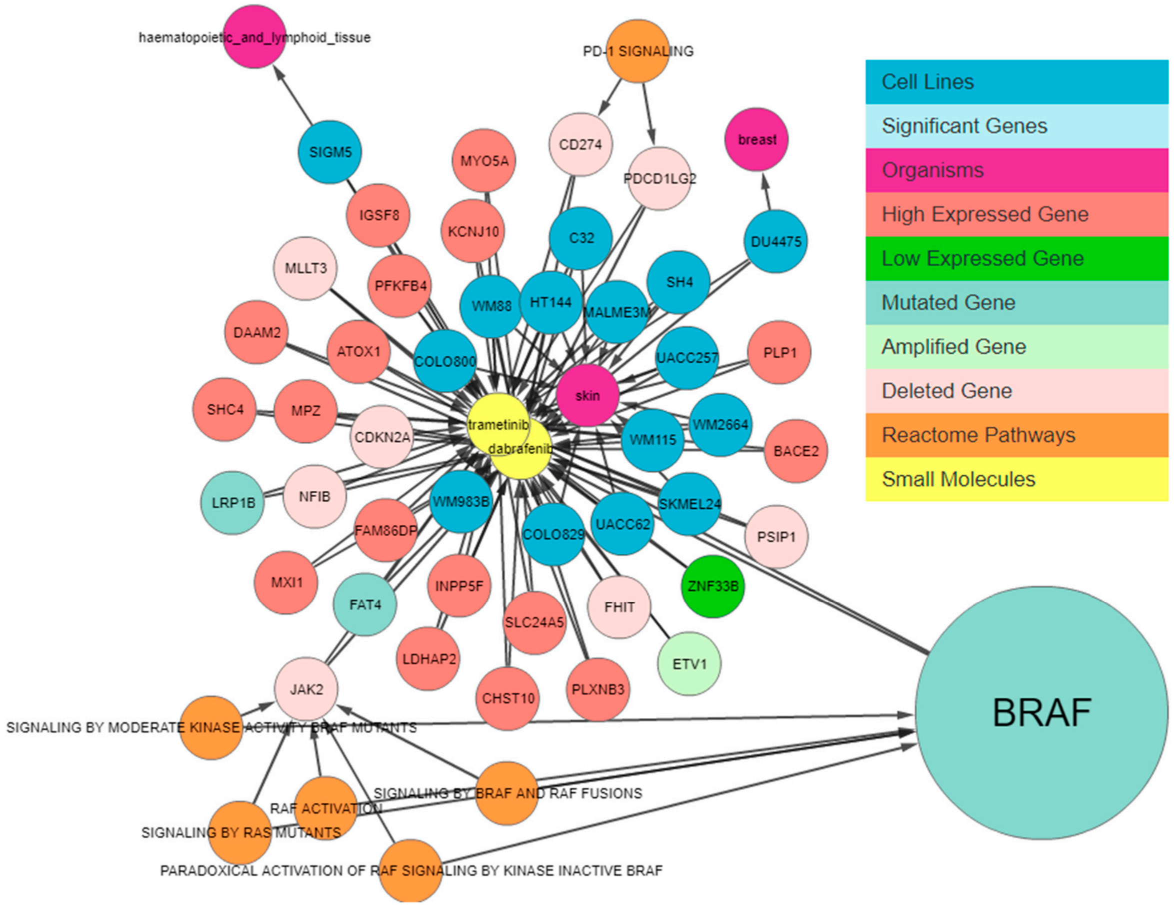

Figure S2: *IPCT output for small molecule user query dabrafenib an trametinib with shared connection enabled. The graph shows all data points connected with dabrafenib and trametinib. Yellow nodes represent small molecules; blue nodes show cell lines sensitive to lapatinib; sky-blue nodes represent significant genes (those with multiple genomic aberrations); green and red nodes represent genes that*

are upregulated and downregulated in the sensitive cell lines, respectively; light green and light red represent the amplified and deleted genes in the sensitive cell lines, respectively; white nodes represent mutated genes; and orange nodes represent the REACTOME pathways of mutated genes.

IPCT User Guide

The IPCT home page is very similar to any other search engine home page that provides users a state forward views to input small molecule and select the filters for the graph generation. Figure S1 and S1A show the IPCT homepage which consists of filters, gene or small molecule selector, database selector and input text box.

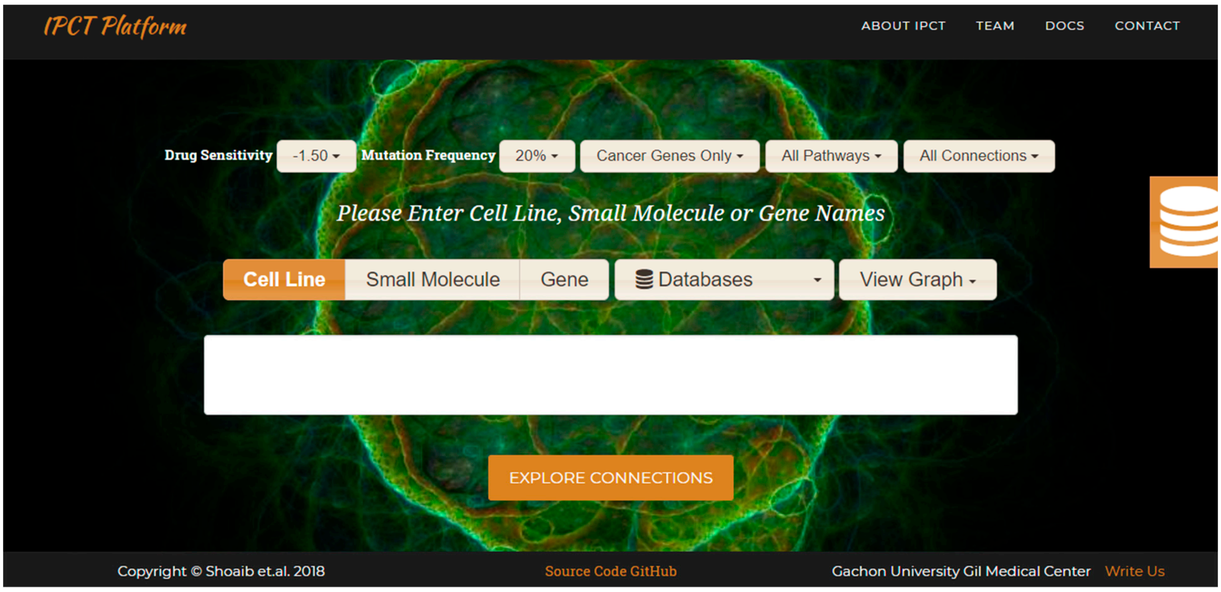

Figure S2: Homepage of Integrated Pharmacogenomic platform of cancer Cell lines and Tissues.

When user enter list of cell lines, small molecules, or the output of IPCT is graph figure S2 shows the IPCT graph displaying window. On the top the screen of genes or small molecules that had contributed towards creation of graph are sown. In the middle of the screen the graph and bottom is general footer.

User can edit the list of genes by clicking on the "edit" button and download the graph by clicking on the "download" button. Upon selecting the edit option screen similar to figure S3 appears which allow users

to alter the list of genes or small molecule as well alters the filtering options as well. Furthermore figure S4, S5 and S6 shows the screen short of various filtering options

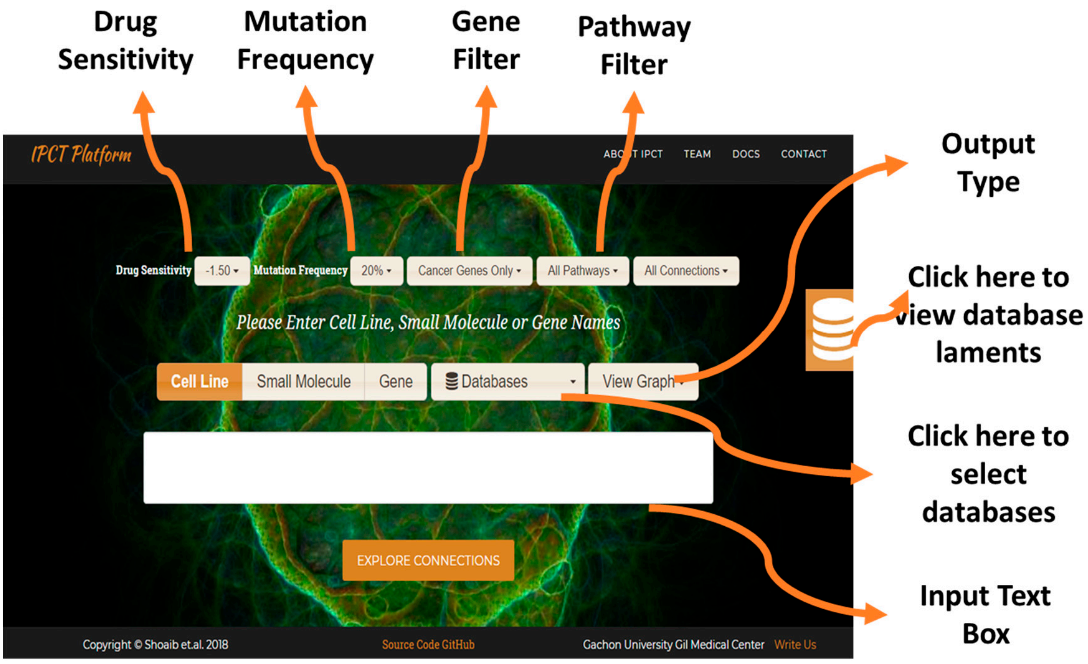

Figure S2A: IPCT components

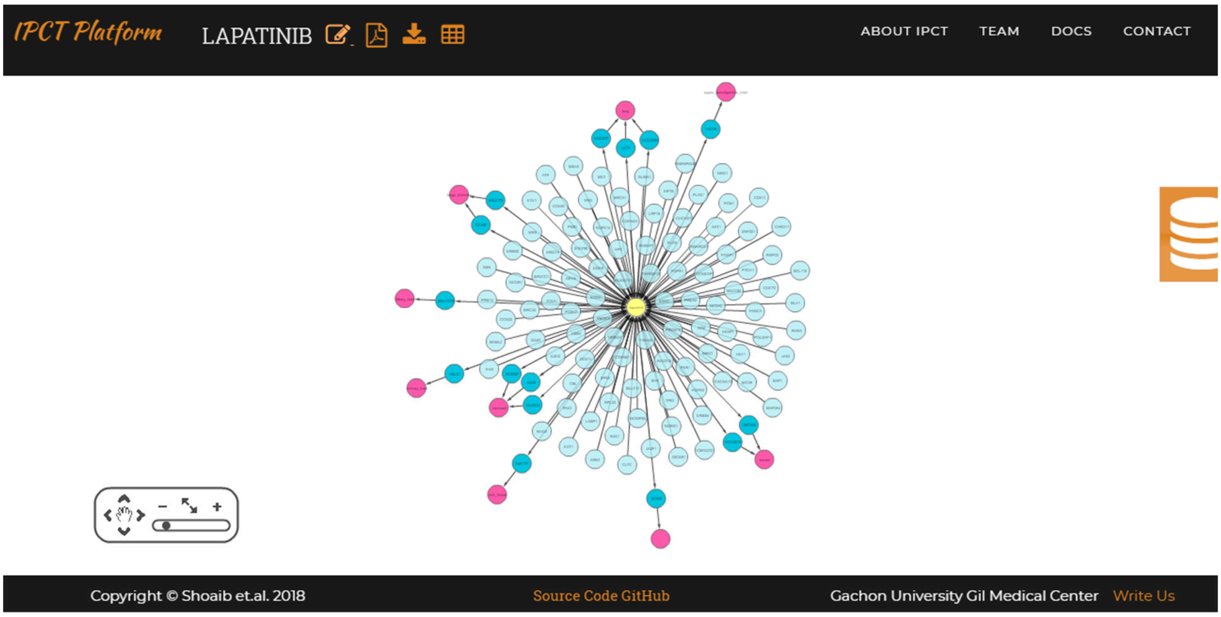

Figure S3 IPCT output windows which includes name of small molecule – "LAPATINIB" in this case –, edit button, and download button and "tabular display" button. The "edit" button can be used to edit list

of small molecules or update filters whereas "download" button can be used to download the graph as PNG file. "Tabular display" button allows users to view the genes and their differential expression conditions in a tubular format.

Grap Filters

In the following we have shown the details about filters andtheir functions along with screen short for each filter.

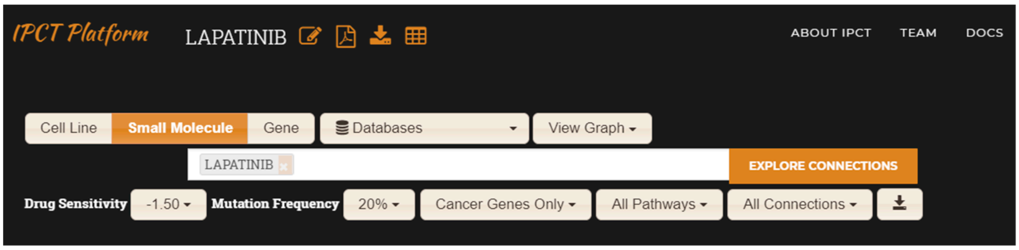

Figure S4: IPCT editing window that appears after clicking the edit button.

Drug Sensltivity FIlter

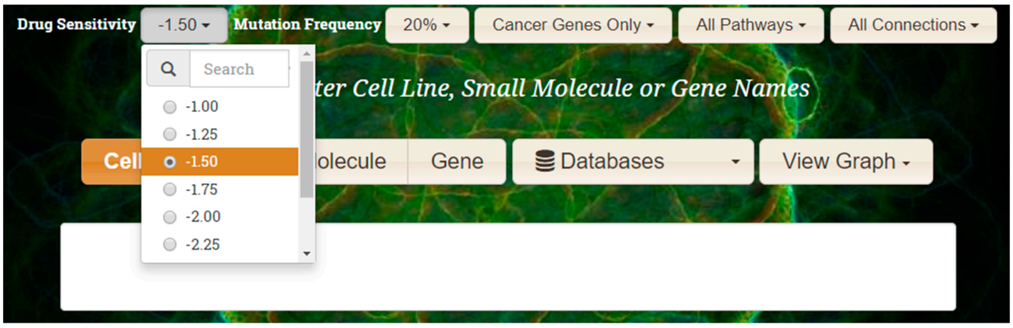

Figure S5: IPCT Drug Sensitivity Filter. Default value for this filter is -1.50 users can decrease the value to increase the sensitivity or increase the value to decrease the sensitivity. If users decrease the sensitivity low sensitive cell lines will also be displayed in the graph and thus the number of cell lines would be increased and vice versa.

Mutation Frequency Filter

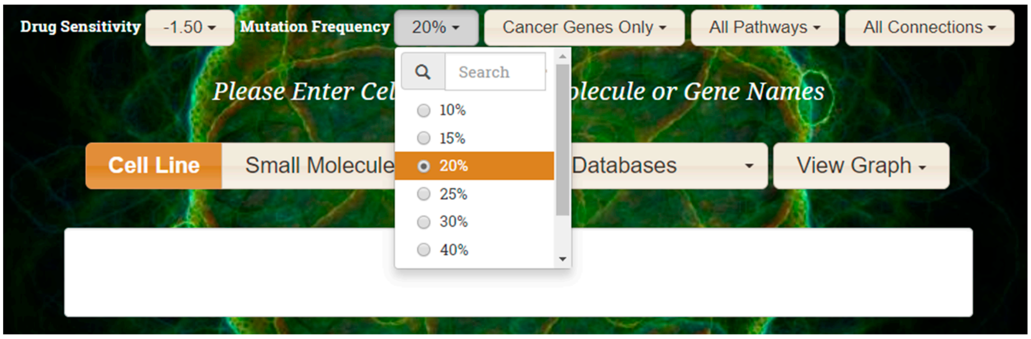

Figure S6: IPCT mutation frequency filter that users can use to specify threshold for displaying mutated genes. IPCT will only output those genes that are mutated mutation frequency greater then given threshold in sensitive cell lines. The default value is 20% which means by default IPCT will only display genes that are mutated in 20% of cell lines.

Mutated Genes Filter

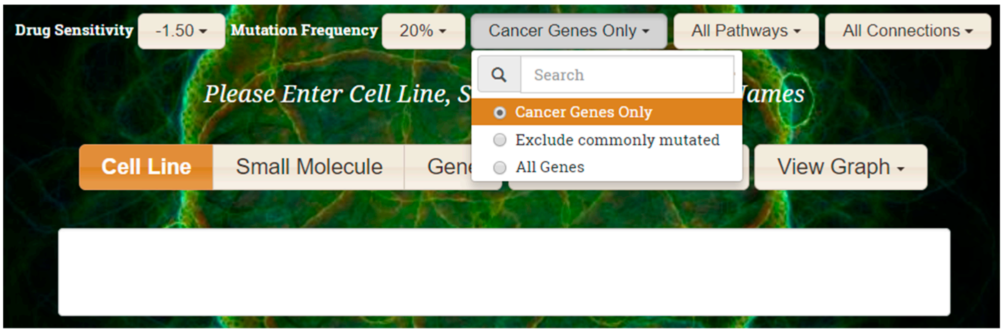

Figure S7: IPCT Gene Filter that allows users to filter list of mutated genes. By default, IPCT will display only cancer genes which are mutated in sensitive cell lines. Using this filter users can either display all genes or limit their search to cancer genes. This filter also allows users to exclude commonly mutated genes i.e. those genes which has mutation in more then 700 cell lines.

Database Selector

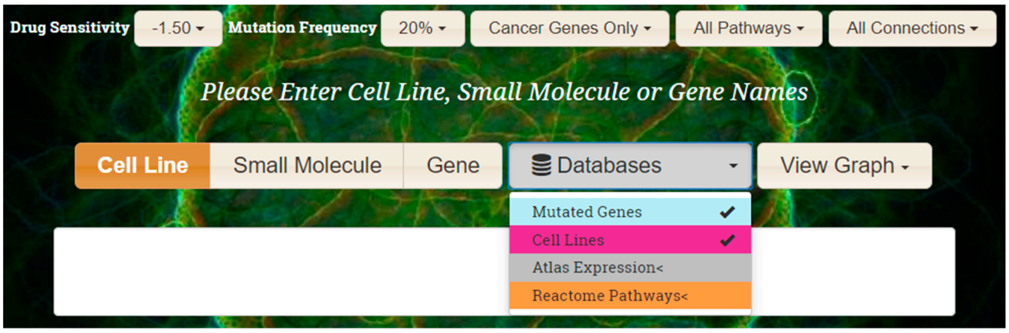

Figure S8: IPCT database selector that allows the users to select the database of their choice. When a user deselected a database the output graph will not contain the database entities from the deselected database.

Other FIlters

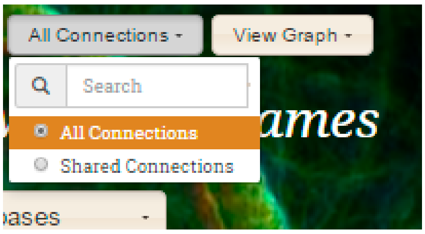

All and shared connections filter

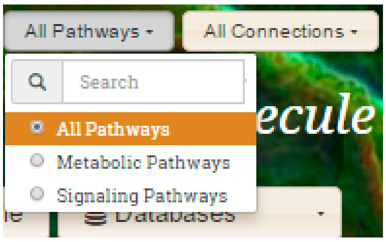

Metabolic and Signaling pathway filter

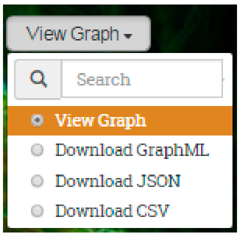

Output Option

Similarly other filter includes (1) "Metabolic" and "Signaling" pathways filter, (2) "All" and "Shared Connections".

Output Options:

IPCT allows users to select their output type. These output types are

- View graph: displays the graph on the user's screen
- Download GraphML: downloads the user's device in the GraphML format–,
- Download JSON: option downloads the user's device as JSON Array–,
- Download CSV: downloads information about graph nodes and edges to the user's machine in CSV format.

Downloading Graph as Image:

In order to save graph as PDF or PNG, JSON, CSV user may click PDF or Download Button after graph is displayed on the screen.

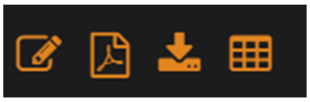

Figure S9: (left right) (1) Edit input, (2) Download PDF, (3) Download Image and (4) Tabular Display.
